# Diseases of Antrum Maxillare

**Published:** 1837

**Authors:** S. P. Hullihen

**Affiliations:** Wheeling, Va.


### Art. II.—Diseases of the Antrum Maxillare.

*Observations on Absess, Purulent Secretion, and Fungous Tumors of the Antrum Maxillare.* By S. P. Hullihen, Wheeling, Va., concluded from January No., page 342.

The terms Fungous and Polypus are generally confounded and applied to all tumors of the Antrum Maxillare, without that attention being paid to the difference which really exists. Indeed Dr. John Bell has labored much to prove that the malignant cancer, like fungous which sometimes attacks the antrum, is at its commencement invariably "moveable, pale and colorless," differing in no particular from the common polypus of the nose, and is almost universally brought on by colds, "attended with violent paroxysms of sneezing, which has never for a moment ceased until the polypus was perfectly formed." (Jno. Bell's Principles of Surgery, vol. 3, page 147.) On page 118 he says:—"Polypus is never mild, nor never malignant; time and the natural growth of the tumor, and the pressure it occasions within the soft and long cells of the nostrils and jaws, must bring every polypus to one invariable form in its last and fatal stage."

That the "moveable pale and colorless" polypus is sometimes produced by colds is very probable; but that pressure on the boney cells of the nostrils and jaws, would in time turn this same "moveable pale and colorless" polypus into a dangerous bleeding fungous tumor, I am not prepared to admit, and will endeavor to show that a fungous growth or tumor does sprout up from ulceration of the lining membrane of the antrum, which is always malignant and has not the slightest analogy to the moveable, pale and colorless polypus of the nose and antrum. The difference in the origin and progression of the fungous and polypus tumor is sufficient to convince the most skeptical that they are two separate and distinct diseases, and unlike each other in every particular. The moveable, pale and colorless polypus, according to Mr. Bell is produced by colds, and sneezed into existence. After the tumour enlarges so as to fill the cavity, a clear transparent lymph is frequently discharged from the nose. After this stage of the disease a watering of the eyes and "toothachey pains" are experienced on the side affected—then an enlargement of the cheek bone and a sanious and fœtid discharge, together with caries of the bones—then deafness, pressure on the brain, frequent hæmorrhage and death.* A fungous tumour of the antrum is always preceeded by a discharge of pus produced by some local irritation. This discharge continues for a shorter or a longer period until the functions of the membrane are destroyed, then small white ulcers appear in patches over the membrane from which small fungosities arise of a deep red color. Soon after this period of the disease, the discharge from the antrum becomes very fœtid and of a thick consistency. As the tumour progresses, sharp pricking pains are experienced in it from time to time until the antrum is entirely filled; then a sensation of great weight or pressure is complained of by the patient. Thus far I have witnessed the progress of this kind of tumour.

In the winter of 1833 Mr. K. of Stark county, Ohio, brought his little son a lad of eight or nine years of age to my office, to consult me in relation to a disease on the left side of his upper jaw. On examining his mouth I found the gums very much swollen and spongy, the palate and roof of his month highly inflamed, and his teeth covered with one continued scale of tarter. On touching the teeth with an instrument, on the side affected, I found them all move together with their alveoli, from the lateral incision back. I inquired whether he had received an injury on that side of his face, and was informed that a Dr. G. had attempted to extract a tooth some seven or eight months before, but had failed; that on the evening of the same day his face had become much swollen and continued so up to the time I saw him. Upon removing the teeth and their alveolar processes, the whole cavity of

* The discharge of clear transparent lymph, the watering of the eyes and the "toothachey" pains on the side affected, I have seen produced by polypus, but the manner a polypus is brought on, and its dreadful destruction of the bones by pressure, its causing deafness--pressure on the brain, frequent hæmorrhagy and death, (having never seen) I copy from Mr. Bell.

antrum was left exposed. After washing out the antrum, I discovered several ulcers in its lining membrane, and two protuberances of a deep red color, one about the size of a pea, the other not so large. I directed his antrum to be washed out frequently with a suitable wash and dismissed him. Two days after I saw him again, and was surprised to find that the protuberances had so enlarged at their bases as to meet, now forming a tumour evidently fungous. The ulcers were slowly enlarging, their edges thickening and of a blueish color. In two or more there was some appearance of fungous. I now applied the lunar caustic to the tumour, and directed the antrum to be washed out with a weak solution of the same. Two days after this I saw my little patient again, and found the main tumour spreading from ulcer to ulcer, and small fungosities sprouting up more or less from all the remaining ones. I now removed the tumour with the knife, and after the bleeding had subsided, which was rather profuse, I applied the lunar caustic very freely to the bleeding surface, and to such places as had the least appearance of fungous. The tumor however returned again and again with redoubled vigor, the child now became weak and sick, the father became alarmed and proposed a consultation, to which I willingly agreed. A consultation was accordingly called and I proposed to use the *actual cautery*. This both shocked and alarmed the consulting physician, a misunderstanding soon followed, and we separated without agreeing upon any plan of treatment. The next day however Mr. K. called upon me again, and requested me to use the actual cautery, if I thought I could thereby destroy the disease effectually. After causing suitable instruments to be made, I called the day following, accompanied by a friend, and found the tumour had filled the whole antrum, producing great pressure upon the surrounding parts. I removed the tumor and applied the cautery. A second application of the cautery, however was found necessary, after which the tumour never appeared again. In two months from this time the antrum was well, in four months the opening was entirely closed, and has remained well ever since. Here then was a tumour that was not produced by a cold, but by a mechanical injury; it did not arise with a violent paroxysm of sneezing, but from an ulcerated surface, it was not at any time a "moveable pale and colorless" polypus, but had a broad base and was of a deep red color; it did not produce pressure on the surrounding parts so as to occasion caries; yet was decidedly malignant. Those who have read Mr. Shiply's case will remember that the tumour did not fill his antrum when first opened, yet it was of a very malignant character, much resembling a tumour termed *Fungous Hæmatodes.* Taking this view of the marked difference in the tumors now in question, one is led to believe that the cases mentioned by Mr. Bell as terminating in caries of the bones, deafness, pressure on the brain, frequent hæmorrhagy and death, were not at their commencement moveable pale and colorless polypi; but bleeding fungous tumours arising from an ulcerated membrane and always malignant. The description he gives of their appearing again and again, after being removed, and spreading from place to place, corresponds precisely with the tumour last named. After removing a tumor he remarks, "I may say my finger was hardly out of the nostril, ere the tumour again began to protrude." And yet he tells us polypus is never malignant; and further that every tumor of the antrum and nose, is at its commencement a "moveable pale and colorless polypus," and that pressure on the bony cells of the nostrils and jaws must bring every such polypus to one invariable form in its last and fatal stage. That he was mistaken in giving those malignant tumours their locality or place of commencement in the nose, is very evident; and more especially in the case of "Cameron," he says, "I found to my inexpressible horror, that every bone and boney cavity was entirely carious, the partition which divides the antrum from the nose was quite destroyed, the polypus occupying the cavity of the antrum," and yet he would have his reader believe that the tumour originated in the nose, and there producing great pressure on the bony cells of the nostril, caused them to become carious; and that finally the tumor had burst into the antrum; a circumstance I do not believe has ever occurred from the pressure of any moveable pale and colorless polypus. It is the opinion however of several surgeons that there is a marked difference in the tumors arising from the schneiderian membrane, yet a clear and correct classification of those tumours, I believe has not been attempted. They are therefore often confounded—the treatment is thereby rendered defective, and consequently a permanent cure of them all is seldom if ever effected. Having now shown the difference in two distinct tumours of the antrum maxillare, I will next proceed to point out in a general manner, the difference that is necessary to be observed in the treatment of the two diseases.

The first and most important step that is to be observed in the treatment of any tumour, is to make a free and large opening into its cavity. Indeed as a general rule, the whole floor of the antrum should be sawed out; then the operator will be enabled to see the full extent of the tumour, and to remove it more effectually. He can also see where to apply his remedies, without injuring the surrounding parts, which is of some importance at least in the treatment of the common polypus tumor. The moveable pale and colorless tumor is generally of a pyriform shape, its attachment is generally small and in the upper corner of the antrum, next to the nasal cavity. Sometimes there are several small polypi, but their attachment is always on or near the partition which separates the antrum from the nose. The best way to remove this kind of tumor, is with a knife made expressly for that purpose, and care should be taken to shave it off down to its very base. The tumour may then be extracted and a piece of sponge saturated in a strong decoction of nutgalls held to the bleeding surface. The hæmorrhage, from this kind of tumor, however, is generally trifling, and never dangerous. After the bleeding has subsided and the antrum is carefully washed out, lunar caustic in the crayon should be applied to the bleeding surface and its immediate edge. The caustic may be applied from time to time, to all suspicious places that may appear, but a second application of the caustic will rarely if ever be found necessary, where the tumor has been properly removed with the knife. Where a tumor is large or of long standing, the lining membrane of the antrum becomes spongy and much out of tone, and should always be attended to. This part of the treatment and the manner an opening into the antrum is usually closed, I will speak of bye and bye.

A fungous tumour makes its appearance in every part of the antrum; it has a large base and is of a deep red color. It is generally spongy and bleeds on the slightest touch. It increases in size very rapidly on being irritated and becomes more malignant as it enlarges. The mere removal of the tumor makes it more dangerous, and the application of lunar caustic, or caustic potash tends only to aggravate the disease. The *actual cautery*, therefore, is the only means that can be resorted to with any hopes of success, and even this unless used promptly, fails to arrest the progress of this "most formidable of all diseases."*

That the cautery may have full effect, the tumour should be carefully and effectually removed by the knife, and the hæmorrhage which is often frightful should be checked. This may be done by filling the antrum for an hour or two, with plugs of lint rolled in the flower of nutgalls. On removing the plugs the *whole internal surface* of the antrum should be seared over, and great pains taken to destroy every vestige of the disease. After the tumour has been completely eradicated, the antrum should be washed out twice a day with soap

---

*I do not know that any one has described the form of a cauterizing iron best calculated for the antrum. The instruments I have been using are two in number; the cauterizing ends are merely round, with a slim probe-like stem about three inches long and fastened into a handle of the same length. The stem can be bent at pleasure, so as to enable the operator to cauterize any part of the antrum. The bulb of the larger cautery is about the size of a large bullet, the other several sizes smaller. The bulb can be heated on a chared coal by means of a lamp and blow pipe, until it is of a white heat, or in other words, until it is as hot as it can be made. It is then to be introduced into the antrum and moved rapidly backwards and forwards, over the remaining portions of the tumour, taking care always to withdraw it while it is yet red hot. When the cautery is thus applied, it disorganizes the parts instantaneously and without producing much pain and causing but little if any inflammation. Whereas a cautery of a lower temperature occasions much pain and inflammation, and has but a trifling effect on the disease.

and water, or other suitable washes, until a new membrane has entirely formed.*

When a membrane has formed anew, after being destroyed or partly so by cautery, it is generally thick and spongy but not painful to the touch, it looks red and throws off a pale blueish pus free from smell. To bring about a healthy action in such a membrane, is always exceedingly difficult, and is by far the most tedious part of the treatment. On the treatment of a spongy or diseased membrane all writers seem to agree. They recommend a solution of zinc, lunar caustic and other astringents to be injected alternately into the antrum twice a day or more, until the membrane is restored to a healthy state. That this course of treatment is proper so far as it goes, I do not pretend to deny, but when we take a pathological view of a spongy membrane and the physiological action necessary for its restoration, something more appears to be necessary at least to bring about a speedy cure. The first object that is to be obtained in the treatment of such a membrane is to promote absorption. The second and of equal importance is to regulate the action of the secreting vessels so as to keep them unloaded, and free as possible from tumefaction, then changing the action of both by deobstruents or local alteratives, which will finally restore the parts to health. Taking this view then of the disease, I have been induced to use *friction*, and with decided success. This is done by brushing the membrane once a day with a small neat brush made for the purpose. Thus stimulated the secreting vessels throw off considerable, the blood vessels still more, and the membrane is thereby reduced to its proper thickness. When thus reduced, the application of astringents, powerfully promote the action of absorbents, so as to bring about that change which is desired, much sooner than can be done by the usual mode of treatment. After the final cure of the membrane is effected, the opening made into the antrum should be closed. This

* A piece of sponge should be kept in the opening made into the antrum, during the progress of its cure, and will answer the double purpose of preventing its closing, and the food from getting into its cavity.

may be done by clasping to the neighboring teeth, a thin gold or silver plate, so formed as to cover the opening and receive its edges. Pressure thus applied will incline the edges inwards and towards each other until they meet. After which they may be pared off and suffered to unite, and the wearing of the plate discontinued.

*February*, 1837.

